# Study protocol: a clinical trial for improving mental health screening for Aboriginal and Torres Strait Islander pregnant women and mothers of young children using the Kimberley Mum's Mood Scale

**DOI:** 10.1186/s12889-019-7845-3

**Published:** 2019-11-14

**Authors:** Emma Carlin, Sarah J. Blondell, Yvonne Cadet-James, Sandra Campbell, Melissa Williams, Catherine Engelke, Des Taverner, Rhonda Marriott, Karen Edmonds, David Atkinson, Julia V. Marley

**Affiliations:** 1 The Rural Clinical School of Western Australia, The University of Western Australia, 12 Napier Tce, PO Box 1377, Broome, WA 6725 Australia; 2 Kimberley Aboriginal Medical Services, 12 Napier Tce, PO Box 1377, Broome, WA 6725 Australia; 3 Apunipima Cape York Health Council, 186 McCoombe Street, PO Box 12045, Bungalow, QLD 4870 Australia; 4 Centre for Indigenous Health Equity Research, Central Queensland University, Cnr of Shields and Lake Streets, Cairns, QLD 4870 Australia; 5 Western Australian Country Health Service – Kimberley Population Health Unit, 4/9 Napier Tce (WA 6725 Locked Bag 525), Broome, WA 6725 Australia; 6 Ngangk Yira: Murdoch University Research Centre for Aboriginal Health and Social Equity, Murdoch University, 90 South Street, Murdoch, WA 6150 Australia; 7 King’s College London, 1 Lambeth Palace Road, Waterloo, London, SE1 7EU UK

**Keywords:** Indigenous, Aboriginal, Torres Strait Islander, Perinatal mental health, Depression, Anxiety, Clinical screening tools

## Abstract

**Background:**

Improving the rates of, and instruments used in, screening for perinatal depression and anxiety among Aboriginal and Torres Strait Islander women are important public health priorities. The Kimberley Mum’s Mood Scale (KMMS) was developed and later validated as an effective and acceptable perinatal depression and anxiety screening tool for the Kimberley region under research conditions. Other regions have expressed interest in using the KMMS with perinatal Aboriginal and Torres Strait Islander women. It is, however, important to re-evaluate the KMMS in a larger Kimberley sample via a real world implementation study, and to test for applicability in other remote and regional environments before recommendations for wider use can be made. This paper outlines the protocol for evaluating the process of implementation and establishing the ‘real world’ validity and acceptability of the KMMS in the Kimberley, Pilbara and Far North Queensland in northern Australia.

**Methods:**

The study will use a range of quantitative and qualitative methods across all sites. KMMS validation/revalidation internal consistency of Part 1 will be determined using Cronbach’s alpha. Equivalence for identifying risk of depression and anxiety compared to a standard reference assessment will be determined from receiver operating characteristic curves. Sensitivity and specificity will be determined based on these cut-points. Qualitative methods of phenomenology will be used to explore concepts of KMMS user acceptability (women and health professionals). Additional process evaluation methods will collate, assess and report on KMMS quality review data, consultations with health service administrators and management, field notes, and other documentation from the research team. This information will be reported on using the Dynamic Sustainability Framework.

**Discussion:**

This project is contributing to the important public health priority of screening Aboriginal and Torres Strait Islander women for perinatal depression and anxiety with tools that are meaningful and responsive to cultural and clinical needs. Identifying and addressing barriers to implementation contributes to our understanding of the complexity of improving routine clinical practie.

**Trial registration:**

The study was registered retrospectively on 15/05/2019 with the Australian and New Zealand Clinical Trial registry (ACTRN12619000580178).

## Background

There is little evidence of the prevalence of perinatal depression and anxiety among Aboriginal and Torres Strait Islander women in Australia [1–3]. Given the higher levels of trauma and distress in the Aboriginal population as a whole, it is understood that rates of perinatal depression and anxiety are also higher than the national average [1–4]. Untreated perinatal mental health disorders can have a severe negative impact on the mother, child and extended family [1, 4, 5]. Specific impacts of perinatal depression and anxiety may include poorer birth outcomes, poorer bonding and attachment between mother and baby, ongoing emotional and cognitive difficulties for children, and enduring (and possibly escalating) mental health disorders for the woman [6–8].

In 2008, the Australian Government launched the National Perinatal Depression Initiative 2008–2013 [9], promoting a universal clinical screening schedule for perinatal depression. The Edinburgh Postnatal Depression Scale (EPDS) [10] was the recommended clinical screening tool. The EPDS was developed in 1987 and is a 10-item depression scale, which also includes a 3-item anxiety subscale. The EPDS, however, has not been validated for use with Aboriginal and Torres Strait Islander women [11]. In the Kimberley, a sparsely populated region in North West Australia, health professionals were concerned that the language and concepts in the EPDS unintentionally disengaged Aboriginal women from the screening process [12]. Consequently, use of the EPDS has been limited among Aboriginal women, and perinatal depression and anxiety are often not identified [12, 13]. In 2013 Gausia et al. [3] reported that Aboriginal women were four times less likely to be screened than non-Indigenous women. Another recent study from Queensland (*n* = 30,468) demonstrated that, after adjustment for socio-demographics, Aboriginal and Torres Strait Islander women were approximately half as likely to have been screened using the EPDS than non-Indigenous women (OR 0.47, 95% CI 0.39–0.56) [14]. Improving the rates of, and instruments used in, screening for perinatal depression and anxiety among Aboriginal and Torres Strait Islander women are important public health priorities.

To initiate improvements in perinatal depression and anxiety screening in the Kimberley region, over 100 Aboriginal women and 70 staff from Kimberley Aboriginal Community Controlled Health Organisations (ACCHOs) and the Western Australian Country Health Service (WACHS) were consulted. The objective of the consultation was to develop a more appropriate way for health professionals to engage Aboriginal women and screen for perinatal depression and anxiety [12]. The consultation resulted in the development of the two-part Kimberley Mum’s Mood Scale (KMMS) (Additional file 1). Part 1 of the KMMS adapts the EPDS using language and graphics determined through the consultation process. Part 2 involves “yarning” [15, 16] as a culturally secure approach for health professionals to explore select psychosocial risk and protective factors with women. The yarning topics were identified through both the consultation process and existing resources [17] relating to good psychosocial care in the perinatal period for Aboriginal and Torres Strait Islander women. The KMMS is designed to be administered by providers of perinatal care including midwives, child health nurses (CHNs), Aboriginal Health Workers (AHWs) and remote area nurses (RANs) who complete the tool alongside and in partnership with Aboriginal and Torres Strait Islander women.

In 2013–14, the KMMS was validated in a study with 91 Aboriginal women from 15 communities and towns in the Kimberley [13]. Overall KMMS risk equivalence for screening for clinically diagnosed depression and anxiety disorders was greater than or equal to moderate (sensitivity 83%; specificity 87%; positive predictive value 68%). During the validation study, participants and their health professionals were asked, using short semi-structured surveys, about their experience with, and the acceptability of, the KMMS. Results from the survey demonstrated the KMMS was well-accepted by the participants and their health professionals. The Aboriginal women who participated in the Kimberley validation study placed high value on patient–health professional rapport and having the time and opportunity to yarn. Completing the tool with a health professional rather than alone was also highly valued.

The findings of the Kimberley validation study [13] have led to endorsement of the KMMS by the Kimberley Aboriginal Health Planning Forum (KAHPF) as the recommended perinatal depression and anxiety screening tool for all Aboriginal and Torres Strait Islander women in the Kimberley region [18]. To better embed the KMMS into routine clinical practice we need to understand ‘real world’ KMMS utilisation/implementation, such as quality, clinical support required and acceptability. We also need to test the validity of the KMMS at a population level, i.e., ‘revalidate’ the KMMS in a larger Kimberley sample via a real world implementation study. Other regions in Australia have also expressed interest in using the KMMS with Aboriginal and Torres Strait Islander women during the perinatal period. Before recommendations for wider use can be made, we need to test for applicability in other regions. This paper outlines the protocol for evaluating the process of implementation and establishing the ‘real world’ quality, validity and acceptability of the KMMS in the Kimberley, Pilbara and Far North Queensland (FNQ) regions of Australia.

## Methods/design

### Objectives

The primary objectives of this study are to 1) evaluate the implementation and real-world performance of the KMMS in the Kimberley; 2) test the validity of the KMMS in the Pilbara and FNQ; and 3) assess the acceptability of the KMMS for Aboriginal and Torres Strait Islander women and their health professionals across all sites, i.e. Kimberley, Pilbara and FNQ.

### Partnerships

The study is a partnership with Apunipima Cape York Health Council (ACYHC) in FNQ; WACHS – Pilbara and Mawarnkarra Health Service (MHS) in the Pilbara region of Western Australia; WACHS – Kimberley, and Kimberley Aboriginal Medical Services (KAMS) and its member services across the Kimberley region of Western Australia.

### Study populations

The target populations for this study are Aboriginal and Torres Strait Islander women who are accessing perinatal care in selected sites in the Kimberley, Pilbara and FNQ, and their health professionals.

Sample frame A: Aboriginal and Torres Strait Islander women (aged 16 years and over) who are at least 6 weeks pregnant or between 7 days and 12 months post-partum, who are not known to be currently acutely mentally unwell and who can provide informed consent.

Sample frame B: Health professionals providing care to perinatal Aboriginal and/or Torres Strait Islander women (as per the specifications of sample frame A), including but not limited to midwives, CHNs, AHWs and RANs.

#### Recruitment

All women who meet the inclusion criteria (sample frame A) and are receiving perinatal care from a partner health service will be invited to participate in the study when they receive routine perinatal screening by perinatal health professionals. Women will be provided with an information sheet and will be required to provide written informed consent prior to participating in the study.

Women are able to withdraw consent at any time and their data will be withdrawn from the study results if they request this.

## Study process

**Fig. 1:**
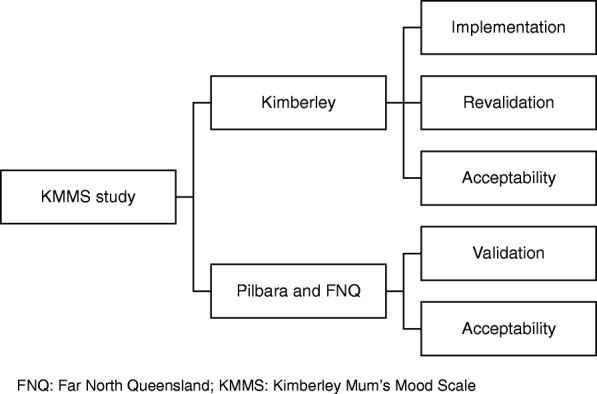
Overview of KMMS and Study Process

The study involves assessment of implementation, validation/revalidation and acceptability of the KMMS as outlined in Fig. 1.

### The KMMS

Part 1 of the KMMS (modified version of the EPDS) includes 10 close-ended questions using regionally appropriate language and graphics. It is an important way to introduce concepts and language around perinatal depression and anxiety, with which women may be unfamiliar [2, 12].

Part 2 is a psychosocial yarning tool [15, 16]. The domains covered in Part 2 include: support; stressors; self-esteem; childhood experiences; relationships; substance use; and social, emotional and cultural wellbeing. Part 2 aims to identify protective and risk factors that may positively or negatively influence a woman’s sense of wellbeing and risk of perinatal depression or anxiety. Health professionals are provided with open-ended prompts to help facilitate the yarn (Additional file 2) [19].

After the KMMS is completed, health professionals then use their clinical judgement and experience to interpret Part 1 and Part 2 to determine a woman’s overall risk of perinatal depression and anxiety. Categories are: low risk (self-care recommended); moderate risk (clinical assessment within 1 week); and high risk (clinical assessment within 72 h). Any concerns regarding a woman’s risk of harm to herself or others (as raised in Part 1, question 10; Additional file 3) requires immediate action in accordance with the health service policies/protocols.

In the Kimberley it is recommended regional practice for all Aboriginal women to be screened with the KMMS (18) with the same frequency and timing as the national EPDS screening schedule [4]. When repeating screening on the same woman during the same perinatal period, a new Part 1 is completed; however, the yarn in the original Part 2 is continued as opposed to starting Part 2 from the beginning. It is recommended practice for those scoring moderate or high risk on the KMMS at any stage to have further clinical assessment by a general practitioner (GP) or mental health professional [18].

#### Training perinatal health professionals

All perinatal health professionals in partner health services will be offered approximately 2–3 h of KMMS training. Training will be divided into three sessions:

- KMMS in-service (50 min) designed to be delivered to all clinical staff. This training will provide information and awareness on perinatal mental health and wellness, provide an overview of the KMMS and ensure that all clinical staff are aware of the KMMS project.
- KMMS administrator training (1.5–2 h) designed to be delivered to health professionals who will be administering the KMMS. This will provide an in-depth explanation of how to use the KMMS, determining the overall risk level and how to recruit and consent women to the study.
- KMMS Diagnosis and Management Tool training (50 min). GPs and mental health professionals will be provided with specialist training in how to use the standard reference assessment, called the KMMS diagnosis and management tool (DMT; Additional file 4). This training will provide an overview of the KMMS screening process, the origins of the DMT and how to use the DMT as part of diagnosis and management of perinatal depression and/or anxiety.

All training will be provided face-to-face or via online classroom workshops. For health professionals involved in the KMMS administrator or DMT training, support and mentoring in using the KMMS tools will be available for the duration of the study.

#### Administering the KMMS

Perinatal health professionals from partner health services trained in the KMMS will consent participants and administer the KMMS instead of the EPDS in accordance with the KMMS guidelines for administration [19]. All women who are assessed as having an overall KMMS risk of moderate or high will be referred for further assessment; where possible this assessment will use the DMT. As part of this study, a proportion of women with an overall KMMS risk assessment of ‘low’ will also be referred for further assessment using the DMT to confirm that the current cut-point between low and moderate is valid.

#### Further clinical assessment

The DMT is the standard reference assessment for all further KMMS clinical assessments. Diagnosis is based on the Diagnostic and Statistical Manual of Mental Disorders (DSM IV) criteria for depressive and anxiety disorders [20] and severity (i.e. low, moderate, high) on the GP Mental State Examination [21]. Participants who are referred for an assessment using the DMT will be assessed as soon as practicable after the KMMS is administered. For those diagnosed with perinatal depression and/or anxiety, a follow-up management plan will be negotiated between the participant and the GP / mental health professional, ensuring appropriate support, referrals and treatment is discussed and agreed upon by both the woman and the health care professional.

### Proposed intervention

The proposed intervention (KMMS) for the three geographical areas is outlined below.

#### Kimberley implementation study

It is recognised that health service implementation is a complex undertaking with many promising research findings failing to translate into improved patient outcomes [22, 23]. Using the Dynamic Sustainability Framework (DSF) [24] we seek to explore and report on the process of implementation. This includes an examination of how the intervention (components, practitioners, outcomes, delivery platform), practice setting (staffing, organisational culture, information systems, business model, training and supervision), and ecological settings (population characteristics, policy, regulation, funding) enable or inhibit implementation of the KMMS into routine clinical care. Specific to the DSF is the expectation that “change is a constant and thus the success of an intervention to be sustained over time lies in the measured, negotiated, and reciprocal fit of an intervention within a practice setting and the practice setting within the larger ecological system” [24]. The DSF posits that ‘fit’ involves the way an intervention is delivered, the characteristics of patients, providers and settings, and the broader system within which health care is situated [24]. The DSF’s recognition and embrace of the constancy of change is compelling and speaks to the realities of the Northern Australian health workforce which is characterised by high turnover, low stability and high use of temporary staffing [25]. Similarly, the emphasis the DSF places on ‘fit’ seems a pertinent starting point for evaluating the process of implementing the KMMS across the geographically large and culturally diverse region of the Kimberley. The learnings and reflections from the process of implementation will be shared with partner health services and the KAHPF, and submitted to relevant peer reviewed journals and national interest bodies contributing to the science of ‘what works’ in the validation and implementation of Aboriginal and Torres Strait Islander health improvement initiatives.

#### Kimberley revalidation study

In addition to studying the implementation process, Kimberley Aboriginal and Torres Strait Islander women offered the KMMS as part of routine care will be invited to participate in the revalidation study. The revalidation will analyse the results of KMMS assessments, any further clinical assessments and support, and the routine care provided when screening for perinatal depression and/or anxiety (see below for detail). During this study, the only modification to the recommended standard of care in the Kimberley will be for a sample of women who score low risk on the KMMS to be offered a follow-up clinical assessment by a GP or mental health professional as part of revalidating the cut-point.

Women who do not consent to the study will continue with usual care and no identifying information will be collected on these women. De-identified information about the number, scores and basic demographics of all KMMS assessments will be requested from partner organisations as part of evaluating the implementation.

#### Pilbara and Far North Queensland validation study

In the Pilbara and FNQ selected health services have agreed to trial the KMMS instead of the EPDS to screen for perinatal depression and anxiety. Partner health services will ask perinatal women for consent to participate in the study. This includes completing the KMMS and providing feedback on the screening tool. All women scoring moderate or high on the KMMS and a sample of women scoring low on the KMMS will be referred for a further clinical assessment using the DMT. As in the Kimberley, the KMMS will be offered in accordance with the national EPDS screening schedule [4] and rescreening will involve completing a new Part 1 and continuing the yarn in the original Part 2.

Women who do not consent to the study can use the KMMS, but no identifying information will be collected on these women.

#### KMMS quality review

Systematic and retrospective investigation of patient medical records has long been used in quality assessment and improvement studies, professional education and training, and as an assessment of patient care initiatives [26–29]. This study will review the quality and appropriateness of KMMS and DMT assessments from all sites via two separate panel processes (Additional files 5 and 6).

The panels will convene every 3 months and will consist of a minimum of four members who have relevant clinical expertise and are sufficiently impartial to the study [29], i.e. health professionals not engaged in the administration of the KMMS or the DMT. The panels will be convened by a member of the research team. A minimum of nine and maximum of 12 files will be selected by the KMMS research team and reviewed by the panel at each meeting. A quota sampling strategy [26] will be used to select the files to ensure the panels are reviewing a spread of KMMS/DMT results, antenatal and postnatal women, health professionals, and health services.

Overall findings from each panel will be disseminated back to partner health services as a quality initiative. Where required, additional training and support will be offered to health services to ensure that Aboriginal and Torres Strait Islander women are receiving appropriate care and support via the KMMS and the medical files and records reflect the service provided.

#### Acceptability of the KMMS

User acceptability is a multi-faceted construct that investigates if and how recipients or administrators of a healthcare intervention consider it to be appropriate [30, 31]. The study will assess key domains of user acceptability (Table 1) using a range of methods including questionaries and qualitative interviewing.

**Table 1:** Kimberley Mum’s Mood Scale user acceptability measures adapted from El-Den et al. [30] and Sekhon et al. [31]

User Acceptability measure	Patient	Staff
Tool Content		
Overall acceptability including views on cultural security	✓	✓
Clarity/understanding (purpose, process, questions, rating scales)	✓	✓
Content and concepts	✓	✓
Structure/format and length	✓	✓
Language, terminology and graphics	✓	✓
Process		
Quality of relationship between patient and administrator	✓	✓
Appropriateness of screening location and timeframe	✓	✓
Perceived effectiveness of the tool in achieving its purpose	✓	✓
Perceived benefits/disadvantages of using the tool	✓	✓
Levels of confidence in using the screening tool	✘	✓
Barriers to use, burden of completing, opportunity costs, workload	✓	✓
Other impacting factors		
Perception of importance of screening for perinatal depression and anxiety	✓	✓
Perceptions of prevalence of perinatal depression and anxiety	✓	✓
Perceptions of availability and adequacy of follow up support and treatment	✓	✓
Satisfaction with training	✘	✓
Information, communication and technology requirements	✘	✓
Familiarity with and number of times using the tool	✓	✓

Women consented to the KMMS study will be asked to complete a 5-item paper-based feedback questionnaire immediately after the administration of their first KMMS. This questionnaire will assess participants’ experience with the KMMS tool, specifically exploring perceptions of tool content (Table 1). Additionally, a subset of women (*n* = 6–12 per KMMS region) will be invited to participate in a face-to-face in-depth interview approximately 3–6 months after administration of their first KMMS. The interviews will be no longer than 1 h in duration and will use a methodological approach of phenomenology to explore experiences of completing the KMMS [30, 31]. Recruitment for in-depth interviewing will occur by following up with those women who expressed interest in an interview by leaving their details on the KMMS feedback questionnaire. Additional measures of purposive sampling such as the study team attending mums and bubs groups or playgroups may result in further recruitment. Participants will provide written consent prior to their involvement in the interviews.

Health professionals will also provide data on the acceptability of the KMMS training and KMMS tool. At the time of training, all health professionals will be asked to complete a paper-based feedback questionnaire. This feedback will relate to the health professional’s experience of the training, including their perceptions of the significance and prevalence of perinatal depression and anxiety in the region in which they work. All health professionals who have been trained and have subsequently administered the KMMS will be asked to complete an online questionnaire 6 months post KMMS administrator training. The questionnaire will assess KMMS use and the health professional’s experience of the KMMS as a feature of clinical care.

Additionally, a subset of KMMS-administrator health professionals (*n* = 6–12 per KMMS region) will be invited to complete an in-depth interview (in person or by phone) 6 or 12 months post administrator training. Recruitment will occur via phone calls to all trained administrators. Interviews will be no longer than 1 h in duration and will explore user acceptability measures identified in Table 1.

All user acceptability data will be anonymous (questionnaries) or de-identified (in-depth interviews) and participants confidentiality will be maintained at all times.

### Outcome measures

Primary outcomes:

- Diagnostic accuracy of the KMMS as a screening tool for perinatal depression and anxiety assessed by comparing results of the KMMS with the DMT.
- Acceptability of the KMMS for all sites assessed primarily through the qualitative methodology of phenomenology supplemented with descriptive statistical analysis.
- Implementation of the KMMS in the Kimberley assessed using a process evaluation methodology framed by the DSF.

Secondary outcomes:

- Patterns of change identified in KMMS risk for women who have more than one KMMS assessment.
- Variations in DMT diagnosis determined with KMMS assessment overall score based on if the DMT was conducted blinded or not.
- KMMS quality and appropriateness identified based on the findings of the KMMS quality review panels.

### Sample size calculation

We plan to recruit an equal number of antenatal and postnatal women. The data from antenatal and postnatal women will be pooled for the main analysis, as there was no significant difference in KMMS or study GP assessments between antenatal and postnatal participants in the Kimberley validation study [13]. If there is no significant difference between the data from the three regions, these data will also be pooled.

For the validation/revalidation study, assuming there is a minimum sensitivity of 83% for screening for perinatal depression and anxiety and prevalence is at least 25% (based on the Kimberley validation study), using calculations by Buderer [32] a sample size of 868 paired KMMS and DMT assessments will detect a two-tailed 95% CI with a maximum width of ±5% (i.e., if sensitivity 83, 95% CI 78–88%). This is at the upper end of reported sensitivities for the EPDS compared to a reference standard assessment (59–100%) [33].

Based on the Kimberley validation study [13], we expect 65% of KMMS assessments to be referred for DMT and that at least 75% of the participants who are referred will have both the KMMS and DMT assessments completed (i.e., require 1780 KMMS to obtain 868 paired assessments). For the secondary analysis to determine changes in KMMS risk, we aim to assess as many participants as possible both antenatally and postnatally. As a conservative estimate, we will assume that at least 60% of participants will have KMMS administered at both time points. Recruiting 890 antenatal women (50% of the 1780 KMMS required) should provide at least 534 paired antenatal/postnatal KMMS assessments. In addition, approximately 356 women will be recruited postnatally to ensure that we have 890 antenatal and 890 postnatal KMMS assessments required for the main sample size calculation. Therefore, we initially aimed to recruit 1246 women (890 in the antenatal period and 356 women postnatally) to provide 868 paired KMMS / DMT assessments and 534 paired antenatal/postnatal KMMS assessments.

The complexity of establishing a partnership project across three remote regional areas has taken longer than anticipated; as such a revised sample size is 700 women across all three regions. Assuming 442 of the 700 women have paired KMMS and DMT assessments, this would allow us to detect a two-tailed 95% CI with a maximum width of ±7% (i.e., if sensitivity 83, 95% CI 76–90%).

### Blinding

Where possible, GPs and mental health professionals using the DMT will be blinded to the original KMMS assessment. Blinding is, however, not practical for busy, under resourced health services [34, 35]. Similarly, blinding does not always represent good care with a patient being asked to tell her story again from the beginning [36]. With respect to the ‘real world’ nature of the study many of the KMMS assessment results will be sent to the GP or mental health professional along with the KMMS referral paperwork, or the results will be viewable via the health service’s electronic medical record system. This study will monitor and assess the convergence or divergence in diagnosis of perinatal mental health disorder(s) based on the GP / mental health professional being blinded or aware of the KMMS assessment results.

### Data management

All information collected from participants is treated as strictly confidential.

Electronic data will be stored in the form of Microsoft Word, REDCap, NVivo 12 (QSR International), Microsoft Excel and Stata (StataCorp) documents. Electronic and digital audio data will be stored on a password protected computer supplied by the Rural Clinical School of Western Australia (RCSWA) or partner health service.

Once data has been obtained, collated and checked, it will be de-identified in a re-identifiable manner, with respect for the preservation of individual privacy. Medical information that is linked with a participant’s identity will not be shared with parties that do not have a direct role in health care for that participant.

Paper based consent forms will be stored under numerical code in a locked filing cabinet only accessible to study personnel. KMMS research staff will transpose any paper information to a secure database using REDCap electronic data capture tools hosted at The University of Western Australia (UWA). These data will be kept for 5 years after last access of data by the research team, after which they will be destroyed. At this time electronic data stored by the researchers will be de-identified in such a manner that it cannot be re-identified, and will be kept for a minimum of 15 years.

### Data monitoring

Data monitoring is a function of the regional Trial Management Group (TMG). This group consist of the Coordinating Principal Investigator (CPI), Research Fellow, other study staff and partner health service representatives. Data monitoring is a standing agenda item at each of the monthly TMG meetings. Any change or alteration from the procedures stated in this study protocol, consent documentation, recruitment process, or other study materials (e.g. questionnaires) are considered to be protocol deviations and will be assessed by the TMG. In the event of an unexpected adverse event related to the study or a significant protocol deviation this group would convene within 5 days to review and assess the concern. The TMG will report any concerns to the supervising Human Research Ethics Committee as per the streamlined reporting scheme recommended by the National Health and Medical Research Council (NHMRC) and Australian Health Ethics Committee [37].

The data stored on the secure REDCap database described above will be randomly assessed to make sure there are no errors in transcribing. Other data will be routinely checked by senior members of the research team.

### Data analyses

#### Quantitative analysis

KMMS validation/revalidation internal consistency of Part 1 will be determined using Cronbach’s alpha [38]. Equivalence for identifying risk of depression and anxiety compared to the DMT will be determined from receiver operating characteristic curves. Sensitivity and specificity will be determined based on these cut-points.

Differences in characteristics between antenatal and postnatal participants, participants who are or are not diagnosed with depressive and/or anxiety disorders, and participants from different regions will be compared using χ^2^ tests for categorical data and Mann–Whitney tests for continuous non-parametric data. Change in KMMS risk (from low to moderate/high, or vice versa) will be compared using McNemar’s test. Descriptive statistical analysis will be used when assessing trends and patterns in user acceptability.

#### Qualitative analysis

User acceptability data will be descriptively and iteratively coded using NVivo11 (QSR International). The coding categories will be reviewed by the research team and Investigators before the data is thematically analysed [39]. Aboriginal and Torres Strait Islander investigators and project staff will be involved in all aspects of qualitative data coding and analysis to ensure that a cultural lens is applied in the coding and analysis of Aboriginal and Torres Strait Islander participants’ data [40–42].

Quality assessments of the KMMS and DMT as per the Quality Assessment Panel will be descriptive using the structured process identified in Additional Information 3 and 4.

The process evaluation [43, 44] will collate, assess and report on KMMS user acceptability data, KMMS quality review data, rates of KMMS and DMT completed across the regions, consultations with health service administrators and management, field notes from the research team, TMG meeting minutes, and training calendars, via the DSF.

## Discussion

The KMMS is a valid and acceptable screening tool for depression and anxiety among Aboriginal women in the Kimberley region under research conditions [13]. It has the potential to have broader geographical application for other Aboriginal and Torres Strait Islander women. Validating clinical screening tools that are acceptable to the needs and context of Aboriginal and Torres Strait Islander patients increases and informs our understanding of acceptable and culturally appropriate clinical care [17, 19, 21–25]. This project is contributing to the important public health priority of screening Aboriginal and Torres Strait Islander women for perinatal depression and anxiety with tools that are meaningful and responsive to cultural and clinical needs. This approach has the potential to improve the uptake of screening [3]. This in turn could lead to better early identification, support and treatment of mental health disorders for Aboriginal and Torres Strait Islander women [4].

Few studies have reported on the process and outcomes of clinical validation and implementation [45, 46]. Real world studies are complex, at times fragmented and require ongoing detailed planning [47–49]. High staff turnover and subsequent institutional amnesia and delays need to be factored into the planning process. Likewise the ongoing impact of colonisation, racism, different world views, cultural values and beliefs, and language need to be considered in the overall process. Real world validation and implementation studies take time and effort, and require a multidimensional and multidisciplinary approach that involves extensive consultation with heath care providers and patients. Identifying barriers and enablers in the process of real world validation and implementation studies contributes to our understanding of the complexity of improving routine clinical practice. Addressing those barriers and enablers enhances the sustainability of the clinical improvements.

## Supplementary information

**Additional file 1.** KMMS Part 1 and 2. KMMS screening tool Part 1 and 2.

**Additional file 2.** Questions for each Psychosocial Domain. Domains with corresponding psychosocial (open ended) questions.

**Additional file 3.** The KMMS Scoring Template. KMMS Part 1 scoring sheet and exploratory questions for question 10 (I think of doing something bad to myself or others).

**Additional file 4.** KMMS Diagnosis and Management Tool. Tool for diagnosis and management of mental health disorders for GPs and mental health professionals.

**Additional file 5.** KMMS Assessment Quality Review Panel. Terms of reference for the panel established to review the quality and reliability of the KMMS.

**Additional file 6.** KMMS Diagnosis and Management Tool Quality Review Panel. Terms of reference for the panel established to review the quality of the KMMS Diagnostic and Management Tool.

## Data Availability

The datasets generated and analysed during this study will not be publicly available due to the possible identifying nature of the participants when their data is viewed in full. The Kimberley, Pilbara and FNQ regions have small populations of perinatal women and we are careful to maintain the confidentiality of our participants. Once data from the study is published, requests for additional data can be made to the corresponding author and will be assessed on grounds of reasonableness. Findings from the study will be disseminated to partners and the public via face to face meetings, plain language summaries and open access peer-reviewed journal articles.

## Abbreviations

ACCHO: Aboriginal Community Controlled Health Organisations
ACYHC: Apunipima Cape York Health Council
AHW: Aboriginal Health Worker
CHN: Child Health Nurse
DMT: Diagnosis and Management Tool
DSF: Dynamic Sustainability Framework
DSM: Diagnostic and Statistical Manual for Mental Disorders
EPDS: Edinburgh Postnatal Depression Scale
FNQ: Far North Queensland
GP: General Practitioner
KAHPF: Kimberley Aboriginal Health Planning Forum
KMMS: Kimberley Mum’s Mood Scale
MHS: Mawarnkarra Health Service
NHMRC: National Health and Medical Research Council
PAHPF: Pilbara Aboriginal Health Planning Forum
RAN: Remote Area Nurse
RCSWA: Rural Clinical School of Western Australia
TMG: Trial Management Group
WA: Western Australia
WAAHEC: Western Australian Aboriginal Health Ethics Committee
WACHS: Western Australian Country Health Service
