# Supplementary material for: Study protocol: a clinical trial for improving mental health screening for Aboriginal and Torres Strait Islander pregnant women and mothers of young children using the Kimberley Mum's Mood Scale
Source: BMC Public Health. 2019 Nov 14;19:1521. doi: 10.1186/s12889-019-7845-3 (PMC6857148; doi:10.1186/s12889-019-7845-3)
Supplement: Supplementary file 3 — Additional file 3. The KMMS Scoring Template. KMMS Part 1 scoring sheet and exploratory questions for question 10 (I think of doing something bad to myself or others). [file 12889_2019_7845_MOESM3_ESM.pdf]

Kimberley Mum's Mood Scale Scoring Template

NAME:
DOB:
DATE:

Part 1 Instructions: Add up to determine total Part 1 score.
Any score > 0 in Question 10 requires further exploration.

Question					SCORE
1. I can sit down and have a good laugh	Yes, always 0	Yes, sometimes 1	No, not much 2	No, never 3	
2. I look forward for good things to happen	Yes, always 0	Yes, sometimes 1	No, not much 2	No, never 3	
3. I blame myself when things go wrong	Yes, always 3	Yes, sometimes 2	No, not much 1	No, never 0	
4. I worry too much and don't know why	Yes, always 3	Yes, sometimes 2	No, not much 1	No, never 0	
5. I feel frightened and shaky a lot	Yes, always 3	Yes, sometimes 2	No, not much 1	No, never 0	
6. I can't handle all the stress or I stress out	Yes, always 3	Yes, sometimes 2	No, not much 1	No, never 0	
7. I feel really no good, like no-one loves me	Yes, always 3	Yes, sometimes 2	No, not much 1	No, never 0	
8. I can't sleep because I am sad or think too much	Yes, always 3	Yes, sometimes 2	No, not much 1	No, never 0	
9. I am so sad I have been crying	Yes, always 3	Yes, sometimes 2	No, not much 1	No, never 0	
10. I think about doing something bad to myself or others	Yes, always 3	Yes, sometimes 2	No, not much 1	No, never 0	

For any response other than "No, never", ask exploring questions. E.g. Who do you think about doing something bad to? Can you tell me more about these feelings and thoughts you are having? When did you have these thoughts? Do you have a plan? What keeps you safe? Comments:

____

____

____

- [ ] Immediate concerns: Follow Kimberley Self-Harm / Suicide Protocol.
- [ ] If no immediate concerns, continue with KMMS Part 2.

TOTAL PART 1 SCORE
