# Supplementary material for: Study protocol: a clinical trial for improving mental health screening for Aboriginal and Torres Strait Islander pregnant women and mothers of young children using the Kimberley Mum's Mood Scale
Source: BMC Public Health. 2019 Nov 14;19:1521. doi: 10.1186/s12889-019-7845-3 (PMC6857148; doi:10.1186/s12889-019-7845-3)
Supplement: Supplementary file 4 — Additional file 4. KMMS Diagnosis and Management Tool. Tool for diagnosis and management of mental health disorders for GPs and mental health professionals. [file 12889_2019_7845_MOESM4_ESM.pdf]

KMMS Diagnosis and Management Tool

Date: ....

Name of Clinician: ....

Study Site: ....

EMR Number: ....

MMEX ☐ CHIS ☐ Communicare ☐

Other ....

MENTAL STATE EXAMINATION	
General appearance behaviour	Perception
Speech	Cognition
Thought: Stream, form, content	Memory
Appetite	Motivation/Energy
Anxiety	Sleep
Mental health family history	Social history
Abuse History: Substance, sexual/ physical (self or other)	Protective factors

AT LEAST ONE OF THE FOLLOWING

Depressed mood ☐	Loss of interest or pleasure ☐

5 OR MORE (including at least 1 from above present in the same 2 week period)

Sleep disturbances ☐	Psycho-motor agitation or delay ☐	Weight loss or gain ☐	Loss of energy ☐
Difficulty concentrating ☐	Recurrent thoughts of death, suicide, harming others ☐	Worthless guilty ☐	Appetite loss ☐

GENERALIZED ANXIETY DISORDER (GAD):

Excessive anxiety or worry ☐	Difficulty concentrating ☐	Restlessness, on edge ☐
Irritability ☐	Easily fatigued ☐	Muscle tension ☐
Sleep disturbance ☐		

POST TRAUMATIC STRESS DISORDER

PAST EXPOSURE - Both must be present

Exposure to violence, abuse, trauma	☐	Responses involved fear, helplessness or horror	☐

RECURRENCE OF

Flashbacks	☐	Avoidance of stimuli	☐
Nightmares	☐	Numbed emotional responses	☐
Psychological distress	☐	Autonomic hyperarousal (outbusts, hypervigilance, startle, panic symptoms)	☐

DIAGNOSIS USING DSM IV CRITERIA

			MILD	MODERATE	SEVERE
Depression	Yes ☐	No ☐	☐	☐	☐
Anxiety	Yes ☐	No ☐	☐	☐	☐
GAD	Yes ☐	No ☐	☐	☐	☐
PTSD	Yes ☐	No ☐	☐	☐	☐

SAFETY (If any 'Yes' answers must follow up with plan)

Increased irritability/anger (getting wild with others)	Yes ☐	Arousal / agitation / aggression	Yes ☐
Increased harmful actions (lashing out at others)	Yes ☐	Dysregulated mood	Yes ☐
Inadvertent harm to others (e.g. dependants, unborn child)	Yes ☐	Substance use — ETOH or other drugs	Yes ☐
Inadvertent harm to self (e.g. neglect)	Yes ☐		

	Immediate	Short term	Long term	No apparent risk
Current level of risk of self harm	☐	☐	☐	☐
Current level of risk of suicide	☐	☐	☐	☐
Current level of risk to others	☐	☐	☐	☐

PROTECTIVE FACTORS

Currently weak, inaccessible	☐	Currently moderate, limited availability	☐	Currently strong, very accessible	☐

MANAGEMENT PLAN

IMMEDIATE	SHORT TERM	LONG TERM

Documented in Clinical Records Yes ☐
