# Supplementary material for: Study protocol: a clinical trial for improving mental health screening for Aboriginal and Torres Strait Islander pregnant women and mothers of young children using the Kimberley Mum's Mood Scale
Source: BMC Public Health. 2019 Nov 14;19:1521. doi: 10.1186/s12889-019-7845-3 (PMC6857148; doi:10.1186/s12889-019-7845-3)
Supplement: Supplementary file 5 — Additional file 5. KMMS Assessment Quality Review Panel. Terms of reference for the panel established to review the quality and reliability of the KMMS. [file 12889_2019_7845_MOESM5_ESM.pdf]

KMMS Assessment: Quality Review Panel

Terms of Reference

1. Purpose

The Panel has been established to review the quality and the reliability of the Kimberley Mum's Mood Scale (KMMS). Outcomes from this meeting will be disseminated back to health services involved in the KMMS implementation study as a quality improvement initiative. Where required, additional training and support will be offered to health services to ensure that Aboriginal and Torres Strait Islander women are receiving appropriate care and support for their mental health and wellbeing during the perinatal period.

*NB patient data will be de-identified.

2. Meeting Responsibilities

The Panel is responsible for reviewing up to 12 files from the region (purposively selected by the KMMS research team)

- The Panel will be provided with Part 1, Part 1 scoring sheet and Part 2 of the KMMS. The Panel will provide independent opinion as to the quality of the KMMS assessment and if the information provided is congruent or not congruent with the health professionals overall risk rating (Appendix A).

- Results from the Panel's discussion will identify themes for staff training and/or ongoing staff and service capacity building.
- The Panel will delegate follow up to the KMMS project team.
- The Panel will monitor and evaluate follow up actions from previous meetings.

3. Frequency of Meetings

The Panel will convene quarterly until the end of the research project in late 2020. The meetings are scheduled for two hours. Video conference facilities will be available.

4. Chair

The Panel will be chaired by the KMMS Research Fellow or delegate.

The role of the Chair will be to:

- set the agenda for the meeting;
- appoint a minute taker;
- ensure the meeting runs according to schedule;
- ensure follow up is actioned in a timely and responsive manner;
- ensure minutes of previous meetings, follow up actions and agendas are sent to the Panel a minimum of two weeks before the meeting;
- The chair has an administrative only role and will not participate in the rating/ review of patient files.

5. Quorum

The quorum will require the Chair, plus 3 other people (including one health professional) for the meeting to proceed.

6. Representation

Panel members will be chosen based on their understanding of Aboriginal and Torres Strait Islander perinatal mental health and the Kimberley health context. People who are actively administrating and/or validating the KMMS are not eligible for the Panel.

Service	Name	Position Title	Email Address

7. Confidentiality

This meeting is confidential and matters must not be discussed outside of the meeting. The exception to this is dissemination of the de-identified areas related to KMMS capacity building for Kimberley health services which will be collated and disseminated by the KMMS Research Team. Where confidentiality is breached, the matter will be handled by the Chair and follow relevant ethical notification procedures.

Appendix A- Quality Review Panel KMMS tool

Study ID _______________

Quality Area			Summary
**Scoring Part 1**	Score tallied	Yes ☐ No ☐	
	Score tallied correctly	Yes ☐ No ☐	
	Score sheet used/included	Yes ☐ No ☐	
	Other:		
**Q.10 response and follow-up**	Response- No, never	☐	
	Response other than No, Never	☐	
	Adequate follow-up/further enquiry and notes if response other than No, Never	☐	
**Part 2 notes**	Overall Detail	Poor ☐ Fair ☐ Good ☐	
	Completeness: All domains accounted for	Sufficient ☐ Insufficient ☐	
	Risk Factors: identified/explored	Sufficient ☐ Insufficient ☐	
	Protective factors: identified/explored	Sufficient ☐ Insufficient ☐	
**Risk Rating identified**	Risk rating marked correctly at bottom of Part 2 (low, moderate, high, immediate)	Yes ☐ No ☐	
**Follow-up actions**	Follow-up actions recorded/noted	Yes ☐ No ☐ Self care ☐	
	Follow-up actions adequate	Yes ☐	

		No<br>□	
**Risk Congruence**	Congruent with information provided	Yes<br>□<br>No<br>□	
	Not enough information provided to confirm/deny congruence	□<br>Insufficient	
**Key elements-feedback**			

Signature Panel Member:
………………………………………………………………………………………..

Date of Panel: ……………………………………………………………………………………………
