# Supplementary material for: Study protocol: a clinical trial for improving mental health screening for Aboriginal and Torres Strait Islander pregnant women and mothers of young children using the Kimberley Mum's Mood Scale
Source: BMC Public Health. 2019 Nov 14;19:1521. doi: 10.1186/s12889-019-7845-3 (PMC6857148; doi:10.1186/s12889-019-7845-3)
Supplement: Supplementary file 6 — Additional file 6. KMMS Diagnosis and Management Tool Quality Review Panel. Terms of reference for the panel established to review the quality of the KMMS Diagnostic and Management Tool. [file 12889_2019_7845_MOESM6_ESM.pdf]

KMMS Diagnosis and Management Tool: Quality Review Panel

Terms of Reference

1. Purpose

The Panel has been established to review the quality of Kimberley Mum's Mood Scale (KMMS) Diagnostic and Management Tool (DMT) assessments. Outcomes from this meeting will be disseminated back to health services involved in the KMMS implementation study as a quality improvement initiative. Where required additional training and support will be offered to health services to ensure that Aboriginal and Torres Strait Islander women are receiving the appropriate care and support via the KMMS DMT.

*NB All patient data will be de-identified.

2. Meeting Responsibilities

The Panel's responsibilities are to review up to 12 DMTs from across all three regions (purposively selected by the KMMS Research Team):

- o The panel will be provided with KMMS DMT but will be blinded to the DMT overall diagnosis and management plan. The Panel will provide an independent expert opinion as to the patient's diagnosis and required management plan based on the detail of the DMT.
- o After each member of the Panel has individually reviewed the patient's DMT the Panel will use consensus decision making to decide the patient's diagnosis or if no diagnosis should be made and key elements of the patient's management plan (Appendix A).
- o The Panel's decision will then be compared to the original KMMS DMT diagnosis and management plan. Differences in risk ratings and key management plan features between the Panel and the original DMT assessment will be discussed and recorded (Appendix B)

- Results from the Panel's discussion will identify themes for staff training and/or ongoing staff and service capacity building.
- The Panel will delegate appropriate follow up to KMMS project team
- The Panel will monitor and evaluate follow up actions from previous meetings.

3. Frequency of Meetings

The Panel will convene quarterly until the end of the research project in late 2020. The meetings are scheduled for approximately two hours. Video conference facilities will be available.

4. Chair

The Panel will be chaired by the KMMS Research Fellow or delegate.

The role of the Chair will be to:

- set the agenda for the meeting;
- appoint a minute taker;
- ensure the meeting runs according to schedule;
- ensure follow up is actioned in a timely and responsive manner;
- ensure minutes of previous meetings, follow up actions and agendas are sent to the Panel a minimum of two weeks before the meeting;
- The chair has an administrative only role and will not participate in the rating/ review of participant files.

5. Quorum

The quorum will require the Chair, plus 3 other members for the meeting to proceed.

6. Representation

Panel members will be chosen based on their understanding of Aboriginal and Torres Strait Islander perinatal mental health and the Kimberley health context. People who are actively administrating and/or validating the KMMS are not eligible for the Panel.

Service	Name	Position Title	Email Address

7. Confidentiality

This meeting is confidential and matters must not be discussed outside of the meeting. The exception to this is dissemination of the de-identified areas related to KMMS capacity building for Kimberley health services which will be collated and disseminated by the KMMS Research Team. Where confidentiality is breached, the matter will be handled by the Chair and follow relevant ethical notification procedures.

Appendix A- **KMMS DMT Panel (diagnosis and management plan blinded)**

Participant ID	Diagnosis (blinded)	Recommended management plan (blinded)	Quality of original DMT notes 0 (poor) to 5 (excellent)

Signature Panel Member: ……………………………………………………………………………..

Date of Panel: ………………………………………………………………………………………..

Appendix B- **KMMS DMT Panel (non blinded)**

**Participant ID**	**Actual diagnosis**	**Actual Management plan**	**Divergence/ convergence of results** (group comments)	**Feedback for clinician**

Signature Panel Chair: ……………………………………………………………………………..

Date of Panel: ………………………………………………………………………………………
